# Experimental and Theoretical Investigations of Out-of-Plane Ordered Nanolaminate Transition Metal Borides: M_4_CrSiB_2_ (M = Mo, W, Nb)

**DOI:** 10.1021/acs.inorgchem.2c03729

**Published:** 2023-03-29

**Authors:** Joseph Halim, Pernilla Helmer, Justinas Palisaitis, Martin Dahlqvist, Jimmy Thörnberg, Per O. Å. Persson, Johanna Rosen

**Affiliations:** † Materials Design Division, Department of Physics, Chemistry and Biology (IFM), Linköping University, SE-58183 Linköping, Sweden; ‡ Thin Film Physics Division, Department of Physics, Chemistry and Biology (IFM), Linköping University, SE-58183 Linköping, Sweden

## Abstract

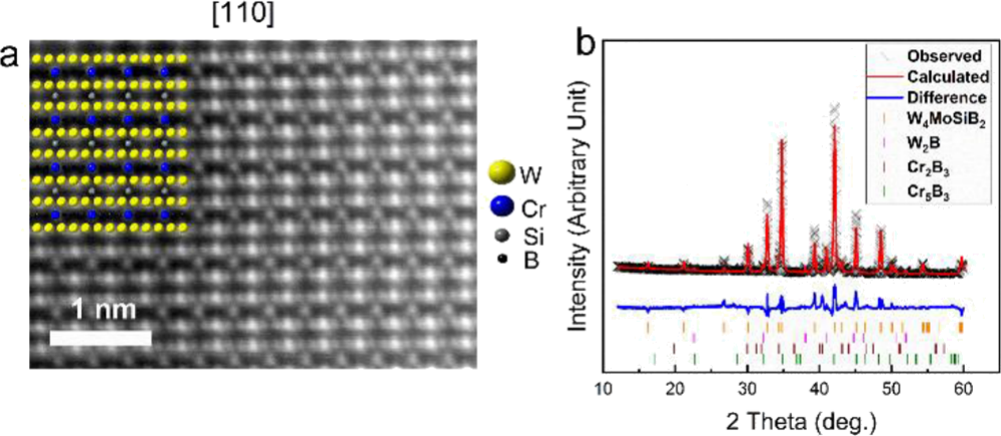

We report the synthesis of three out-of-plane chemically ordered quaternary transition metal borides (*o*-MAB phases) of the chemical formula M_4_CrSiB_2_ (M = Mo, W, Nb). The addition of these phases to the recently discovered *o*-MAB phase Ti_4_MoSiB_2_ shows that this is indeed a new family of chemically ordered atomic laminates. Furthermore, our results expand the attainable chemistry of the traditional M_5_SiB_2_ MAB phases to also include Cr. The crystal structure and chemical ordering of the produced materials were investigated using high-resolution scanning transmission electron microscopy and X-ray diffraction by applying Rietveld refinement. Additionally, calculations based on density functional theory were performed to investigate the Cr preference for occupying the minority 4*c* Wyckoff site, thereby inducing chemical order.

## Abstract 2

We report the synthesis of three out-of-plane chemically ordered quaternary transition metal borides (*o*-MAB phases) of the chemical formula M_4_CrSiB_2_ (M = Mo, W, Nb). The addition of these phases to the recently discovered *o*-MAB phase Ti_4_MoSiB_2_ shows that this is indeed a new family of chemically ordered atomic laminates.

## 1 Introduction

Transition metal carbides and borides are known for their technological importance as electrically conductive refractories with high hardness.^1,2^ When interleaved by A-group elements such as Al or Si, they form atomically layered structures combining both ceramic and metallic properties. The ternary transition metal carbides mainly form the so called MAX phases, with the general formula M_*n*+1_AX_*n*_, where M is the transition metal and X is C and/or N arranged in a hexagonal structure of *P*6_3_*/mmc* symmetry.^3^ Ternary transition metal borides, on the other hand, come in different structures and chemical formulae, such as the orthorhombic structures M_*n*+1_AB_2*n*_ (*n* = 1–3),^4,5^ MAB^6^ and M_4_AB_4_,^7^ the hexagonal structure M_2_AB_2_,^8^ and the tetragonal M_5_AB_2_ (T2).^9^ Most of the MAB phases have Al as the A element^10^ except for the hexagonal M_2_AB_2_ (Ti_2_InB_2_)^11^ and the tetragonal M_5_AB_2_, also called T2 phases, (Mn_5_SiB_2_ and Fe_5_SiB_2_).^12^ In 1957, the first two members of the T2 phases, Mo_5_SiB_2_ and W_5_SiB_2_, were reported by Nowotny et al.^9^ followed by the discovery of Fe_5_SiB_2_ and Mn_5_SiB_2_ in 1960.^13^ More recently, interest in T2 phases has been reignited for their chemical variety, where the M element can be Mo, Mn, Fe, Cr, W, Ta, Co, and Nb, while the A element can be Si, S, P, and Ge.^9,13−19^ This has enabled them to possess various attractive properties such as high oxidation resistance,^20^ near isotropic thermal expansion,^21^ and excellent elastic properties^22^ in addition to magnetic^14^ and superconductive properties.^19^

To expand the chemistry of the MAB phase family and include more transition metals, several attempts have been made to synthesize quaternary MAB with two M elements as solid solutions. Hirt et al.^23^ studied the effect of Co substitution on the magnetic properties of Fe_2_AlB_2_. They reached up to 15 atom % Co in (Fe_1–*x*_Co_*x*_)_2_AlB_2_ using spark plasma sintering, which decreased the Curie temperature from 290 to about 200 K, rendering it as a promising magnetocaloric material. Chai et al.^24^ studied the magnetic properties of the (Fe_1–*x*_Mn_*x*_)_2_AlB_2_ system. Furthermore, Hanner et al.^25^ reported the synthesis of quaternary solid solutions of (Mn_1–*x*_Cr_*x*_)_2_AlB_2_ and (Mn_1–*x*_Cr_*x*_)_3_AlB_4_ phases, while Okada et al. synthesized the quaternary solid solutions (Cr_1–*x*_Mo_*x*_)AlB and (W_1–*x*_Mo_*x*_)AlB.^26^

Chemical ordering in MAB phase alloys has until recently not been reported. Inspired by the discoveries of chemical ordering in MAX phases, in the form of both in-plane ordering, *i*-MAX,^27^ and out-of-plane ordering, *o*-MAX,^28^ evidence was presented for a family of quaternary metal borides with in-plane chemical ordering, *i*-MAB.^29^ In particular, it was found that adding Sc or Y as the M element in Mo_2_AlB_2_, for a ratio of Mo to Sc/Y of 2:1, induces in-plane ordering in the form of Mo_4/3_M′_2/3_AlB_2_, where M′ is Sc or Y.^30^ By selectively etching Al and Sc/Y from the *i*-MAB phase, the first two-dimensional metal boride was discovered as single sheets of Mo_4/3_B_2–*x*_T_*z*_ (boridene).^30,31^ Similarly, by adding Ti to Mo_5_SiB_2_ (a T2 phase) in a ratio of Ti:Mo equal to 4:1, out-of-plane chemical ordering was established, creating the *o*-MAB phase Ti_4_MoSiB_2_,^32^ while using the molten salt (ZnCl_2_) method resulted in the conversion of Ti_4_MoSiB_2_ to single sheets of 2D TiO_*x*_Cl_*y*_.^32^

Recently, a systematic theoretical approach was implemented by Dahlqvist et al.^33^ to predict the stability of *o*-MAB phases in the form of M_4_M′AB_2_ with *M* from Groups 3 to 9 and *A* = Al, Si, P, Ga, Ge. Guided by this and previous work,^32,33^ we present herein the synthesis and characterization of three novel *o*-MAB phases: Mo_4_CrSiB_2_, W_4_CrSiB_2_, and Nb_4_CrSiB_2_, overcoming the challenge of including Cr in Si-based T2 MAB phases. Chemical ordering is confirmed, and Cr is found to reside in Wyckoff position 4*c*. These findings are discussed in the light of results from density functional theory (DFT) calculations.

## 2 Experimental Details

### 2.1 Materials and Synthesis Parameters

Synthesis conditions and parameters are presented in detail in the SI. In short, all phases were synthesized by mixing their corresponding elemental powders (See Table S1) with the ratio of the desired phases, then cold pressed and placed in alumina crucibles and inserted in an alumina tube furnace. The holding temperature and time were 1700 °C and 60 min, respectively. Specific synthesis details for all phases are listed in Table S2. After furnace cooling, the sintered samples were crushed into powders using a mortar and pestle followed by sieving through a 450-mesh sieve.

### 2.2 Characterization Techniques

The structure and weight percentage of the phases present in the produced samples were characterized using X-ray diffraction (XRD) of the powders, by filling a groove of dimensions 20 x 20 x 1 mm^2^ in a glass holder. The measurements were conducted using a PANalytical diffractometer equipped with a Cu K_α_ radiation source (step size = 0.0084° 2θ and time per step = 32 s). The divergence slits and receiving slit of 1/2° and 5 mm, respectively, were used along with a Ni beta filter. To obtain the structural parameters and weight percentages of the phases in a sample, Rietveld refinement of the XRD pattern was performed using *FullProf.* code^34,35^ using peak shape pseudo-Voigt #7. The refined parameters were: five background parameters, scale factors (from which the phases’ weight percentage was obtained), lattice parameters, *X* and *Y* profile parameters, and atomic positions for all the phases, in addition to the global isotropic thermal displacement parameter and asymmetry parameters (the asymmetry parameters are used for asymmetry correction when the peak shapes: pseudo-Voigt #5 or #7 are used, the parameters are four independent asymmetry correction coefficients) for the major phases. The dependency of M (Mo, W or Nb)/Cr site mixing on the refinement was assessed by evaluating χ^2^ for 25, 50, and 75% occupancy on each crystallographic metal site, and the obtained results were only used if the refinement reduced χ^2^ by at least 10% compared to 100% occupancy. If not, site mixing was allowed, and the occupancies were fixed during the refinement.

The microstructure and chemical composition were obtained by scanning electron microscopy, (SEM) (LEO 1550), combined with energy dispersive X-ray spectrometry (EDX). The EDX measurements were acquired from at least 15 particles, all containing M, Cr, and Si in a ratio close to 4:1:1, respectively. Particles that have a stoichiometry close to the secondary phases identified by the XRD Rietveld refinement were excluded. To resolve the atomic structure and ordering of the out-of-plane ordered phases, high-angle annular dark-field scanning transmission electron microscopy (HAADF-STEM) imaging and EDX analysis were performed using the Linköping’s double-corrected FEI Titan^3^ (S)TEM electron microscope operated at 300 kV. Selected area electron diffraction (SAED) patterns were recorded using a FEI Tecnai G2 TEM operated at 200 kV. The HAADF signal intensity of the atomic columns in the HAADF-STEM images are considered as directly interpretable scaling with the atomic number (∼*Z*^1.7^).^36^

### 2.3 Computational Details

The computational bonding analysis was performed using the theory of crystal orbital Hamilton population (COHP) as implemented in LOBSTER.^37−41^ The analyzed structures were relaxed using DFT^42,43^ as implemented in the Vienna ab-initio simulation package (VASP),^44−47^ using the Perdew–Burke–Ernzerhof functional.^48^ An energy cutoff of 400 eV was used for the plane wave expansion, and the structures were sampled with a *k*-point density of at least 0.1 Å^–1^, corresponding to *k*-point meshes of 12 × 12 × 7 or 11 × 11 × 6 for the different structures, depending on the specific cell parameters. The atomic positions were relaxed until a force convergence of 0.005 eV Å^–1^ was reached, and the total electronic energy was within 10^–6^ eV per atom. The projected augmented-wave method^49,50^ was used for treating the core-electrons, with semicore p- and s-electrons considered as valence electrons for the transition metals Cr, Mo, Nb, and W. Including the semicore electrons resulted in a smaller charge spilling when mapping the electron density obtained from VASP in a plane wave basis to the localized atomic wavefunctions needed for the COHP analysis.

## 3 Results and Discussion

**Figure 1:**
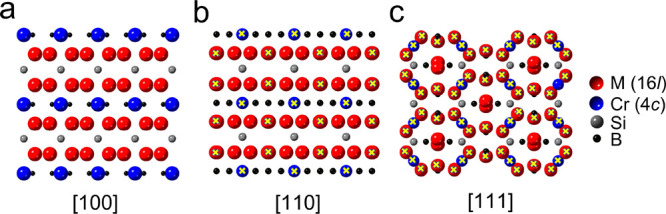
Crystal structure of chemically out-of-plane ordered MAB phases, M_4_CrSiB_2_ (M = Mo, W, Nb), shown for three zone axes: (a) [100], (b) [110], and (c) [111]. Atoms with a yellow cross in (b) and (c), as well as all metal atoms in (a) (red and blue), have an equivalent number of atoms in their corresponding atomic columns along the shown zone axes. Hence, they can be used to conclude chemical ordering based on contrast in STEM images due to the atomic mass difference between M and Cr.

The ternary T2 phases, M_5_AB_2_, have a tetragonal crystal structure of *I*4/*mcm* symmetry, where the M element occupies two Wyckoff sites, 16*l* and 4*c*. The transformation of the M_5_AB_2_ phases into the out-of-plane ordered M_4_M′AB_2_ leads to the occupation of M and M′ at Wyckoff site 16*l* and 4*c*, respectively. Such chemical ordering of the major transition metal (M) and minor transition metal (M′ = Cr) can be verified experimentally using high-resolution STEM imaging, from the zone axis [100] and from the atoms marked with a yellow cross from zone axes [110] and [111], shown in Figure 1. Any observed contrast between the M and Cr atoms in the STEM images can then be linked to the atomic mass differences between the two metals, as the atomic columns along these zone axes have the same number of corresponding atoms in them.

**Figure 2:**
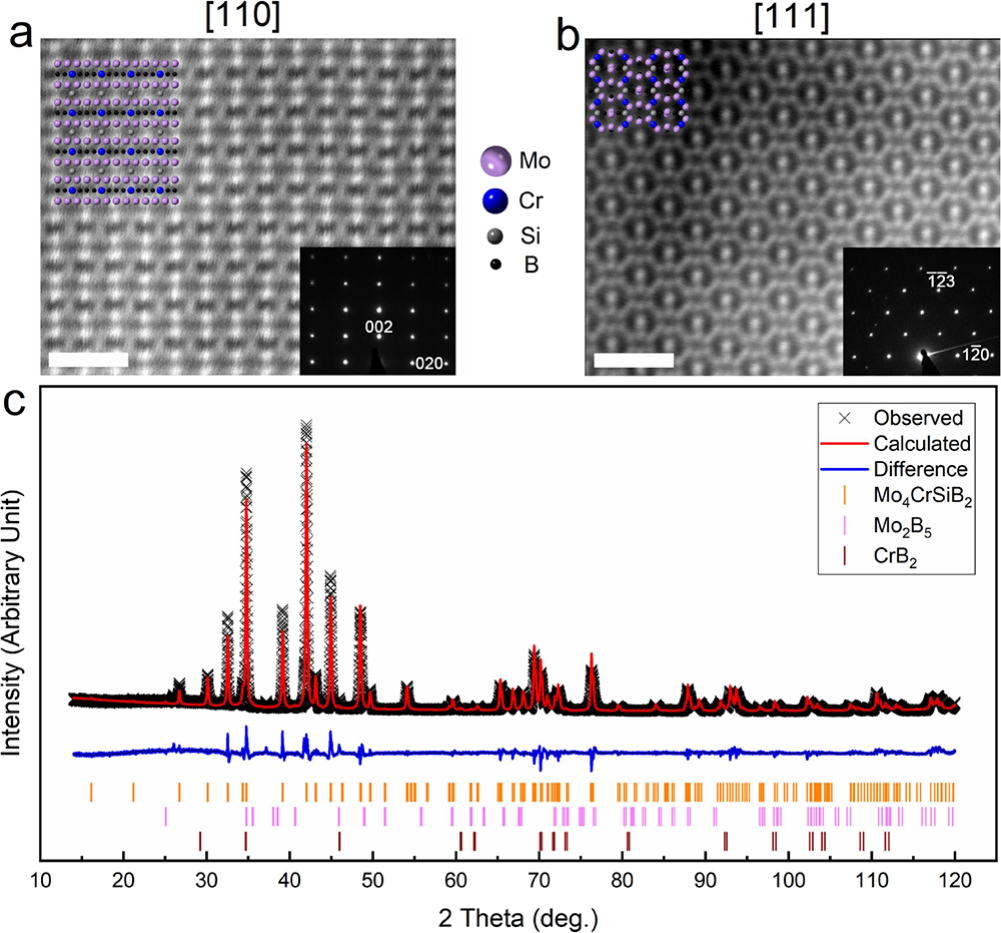
(a, b) STEM images of Mo_4_CrSiB_2_ along the zone axes (a) [110] and (b) [111] with the corresponding SAED shown as insets. Crystal structure for each zone axis is overlaid on its respective STEM image. (c) XRD pattern of the Mo_4_CrSiB_2_ sample showing the measured pattern (black crosses), Rietveld generated pattern (red line), and the difference between both patterns (blue line). The orange, pink, and dark red ticks represent the peak positions of the phases Mo_4_CrSiB_2_, Mo_2_B_5_, and CrB_2_, respectively. Scale bar in (a, b) is 1 nm.

The structure of Mo_4_CrSiB_2_ is shown in the STEM images in Figure 2a,b along the zone axes [110] and [111], respectively. The ordering of Mo and Cr is evident from both zone axes where Mo is the brightest (heaviest), while Cr is less bright. Si and B are too light to be visible in the present images. A potential explanation as to why M in M_5_SiB_2_ can be replaced by Cr at the minority site 4*c* but not at the majority site 16*l*, is presented below in the theory section. The crystal structure, obtained from the refinement of the XRD data (Figure 2c and Table 1) and overlaid on both STEM images, shows an identical atomic arrangement. Furthermore, the SAED shown in the insets of Figure 2a,b confirms the T2 tetragonal structure of *I*4/*mcm* symmetry of the Mo_4_CrSiB_2_.

**Table 1:** Rietveld Refinement Results for the Mo_4_CrSiB_2_, W_4_CrSiB_2,_ and Nb_4_CrSiB_2_ Phases Based on the XRD Patterns Shown in Figures 2c and 3b,d, Respectively^a^

	Mo_4_CrSiB_2_	W_4_CrSiB_2_	Nb_4_CrSiB_2_
space group	*I*4/*mcm*	*I*4/*mcm*	*I*4/*mcm*
cell parameters	*a* = *b*: 5.939(5) Å *c*: 11.016(4) Å	*a* = *b*: 5.942(3) Å *c*: 10.948(3) Å	*a* = *b*: 6.109(1) Å *c*: 11.547(2) Å
	α = β = γ = 90.0°	α = β = γ = 90.0°	α = β = γ = 90.0°
M 16*l*	Mo [0.16616(9) *x* + 1/2 0.13855(7)]	W [0.16443(11) *x* + 1/2 0.13929(8)]	Nb [0.17106(11) *x* + 1/2 0.13813(8)]
Cr/M 4*c*	[0.0 0.0 0.0]	[0.0 0.0 0.0]	[0.0 0.0 0.0]
	occupancy: Cr = 3.228(3) Mo = 0.772(3)	occupancy: Cr = 4 W = 0.0	occupancy: Cr = 4 Nb = 0.0
Si 4*a*	[(0.0 0.0 0.25]	[(0.0 0.0 0.25]	[(0.0 0.0 0.25]
B 8*h*	[(0.34764(198) *x* + 1/2 0.0]	[(0.35605(81) *x* + 1/2 0.0]	[(0.37423(210) *x* + 1/2 0.0]

a Occupancy for the three phases in 16*l* is fixed to 100% M.

SEM images showing the morphology of Mo_4_CrSiB_2_ particles are found in Figure S1a. The chemical composition obtained from EDX in SEM (Table S3) and EDX in TEM (Table S4) is consistent with the following relative elemental atomic percentages: Mo = 68, 70 at. %, Cr = 15, 14 at. %, and Si = 17, 16 at. %, respectively, which are close to the ideal molar ratio of 4:1:1.

The XRD pattern shown as black cross symbols in Figure 2c is obtained from a sample with initial elemental ratios corresponding to a stoichiometry of Mo_4_CrSiB_2_. The red line together with the blue line represent the calculated pattern obtained from the Rietveld refinement analysis, and the difference between the experimental and calculated XRD patterns, respectively. The major phase in the sample was found to be Mo_4_CrSiB_2_ of ≈98.4 wt %, together with a small amount of Mo_2_B_5_ and CrB_2_. The lattice parameters *a* and *c*, calculated from the refinement, were 5.939(5) and 11.016(4) Å, respectively. These values are in agreement (within 1%) with those calculated theoretically as listed in Table S6. It is worth noting that according to the Rietveld refinement, there is a site mixing at the 4*c* site, in other words there is ∼20% occupancy of Mo in the Cr occupied site. However, Mo atoms occupy 100% of the 16*l* site. A comparison to the other *o*-MAB phases as well as the ternary counterparts is found below. The detailed refinement results are found in Tables 1, S2, and S5.

**Figure 3:**
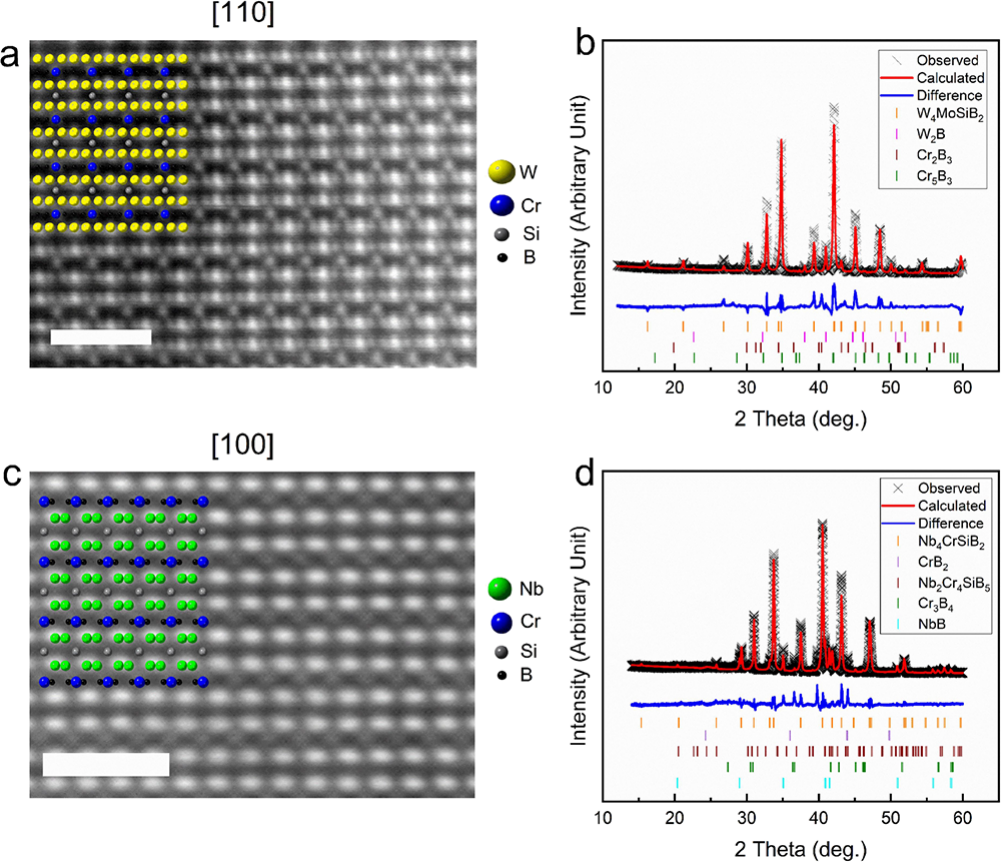
(a) STEM image of W_4_CrSiB_2_ along the [110] zone axis. Crystal structure viewed along this zone axis is overlaid on the STEM image. (b) XRD pattern of the W_4_CrSiB_2_ sample showing the measured pattern (black crosses), Rietveld generated pattern (red line), and the difference between both patterns (blue line). Orange, purple, dark red, and green ticks represent the peak positions of the phases W_4_CrSiB_2_, W_2_B, Cr_2_B_3_, and Cr_5_B_3_, respectively. (c) STEM image of Nb_4_CrSiB_2_ along the [100] zone axis. Crystal structure viewed along this zone axis is overlaid on the STEM image. (d) XRD pattern of the Nb_4_CrSiB_2_ sample showing the measured pattern (black crosses), Rietveld generated pattern (red line), and the difference between both patterns (blue line). Orange, purple, dark red, green, and turquoise ticks represent the peak positions of the phases Nb_4_CrSiB_2_, CrB_2_, Nb_2_Cr_4_SiB_5_, Cr_3_B_4_, and NbB, respectively. Scale bar in (a, c) is 1 nm.

The STEM images of W_4_CrSiB_2_ and Nb_4_CrSiB_2_ are depicted in Figure 3a,c, respectively. Similar to the Mo_4_CrSiB_2_ sample, the STEM images for the W_4_CrSiB_2_ phase (zone axes [110] and [111], shown in Figure 3a and S2b, respectively) show the ordering of W and Cr atoms, as W is brighter (heavier) than Cr. The crystal structure overlaid on the STEM images (Figure 3a) and the SAEDs shown as insets in Figure S2a,b confirm the T2 tetragonal structure with *I*4/*mcm* symmetry of the W_4_CrSiB_2_ phase. Similar observations made for W_4_CrSiB_2_ and Mo_4_CrSiB_2_ may be correlated to Mo and W being in the same group (VI) in the periodic table of elements, i.e., having a similar valence electron configuration.

The morphology of the W_4_CrSiB_2_ particles is shown in the SEM image in Figure S1b. The average relative atomic percentages of W, Cr, and Si, obtained from SEM–EDX on individual particles (tabulated in Table S3) were found to be 70, 16, and 14 at.%, respectively. These values are close to the ideal molar ratios of 4:1:1 for W:Cr:Si, respectively. The XRD pattern for the sample with initial elemental ratios corresponding to the stoichiometry of W_4_CrSiB_2_ is shown in Figure 3b for a selected 2θ range and in Figure S2c for the full 2θ range. The black cross symbols, red line, and blue line represent the obtained XRD pattern, the calculated pattern obtained from the Rietveld refinement analysis, and the difference between them, respectively. According to the refinement, the sample contains ≈90 wt % of the main phase, W_4_CrSiB_2_, while the rest belongs to the binary phases W_2_B, Cr_2_B_3_ and Cr_5_B_3_. The obtained lattice parameters, *a* = 5.942(3) Å and *c* = 10.948(3) Å, are within 1.3% of the theoretically predicted value in Table S6. A comparison to the other *o*-MAB phases as well as the ternary counterparts is found below. Further refinement results are tabulated in Tables 1, S2, and S5.

For the Nb_4_CrSiB_2_ phase, the STEM images along the [100] and [111] zone axes are shown in Figures 3c and S3b. In particular the [100] orientation shows the chemical ordering of Nb and Cr atoms, evident from the difference in brightness that corresponds to the difference in atomic mass. The crystal structure along with the atomic arrangement are shown by the schematic structure overlaid on both STEM images. It should be noted that Nb is an element in group V of the periodic table of elements, and as such has a different valence electron configuration compared to Mo and W. Still, it is found in the chemically ordered structure of an *o*-MAB phase, just like W_4_CrSiB_2_ and Mo_4_CrSiB_2_. Being in the same period as Mo (the majority element of a highly stable o-MAB phase, see above), one can expect a size of Nb being suitable for occupation of the majority site 16*l*, just like for Mo.

The morphology of Nb_4_CrSiB_2_ is shown in the SEM image presented in Figure S1c. The relative amount of Nb:Cr:Si, obtained from SEM–EDX, corresponds to 67, 17, and 16 atom %, respectively, and is shown in Table S3. Once again, these values closely match the ideal 4:1:1 molar ratios of Nb:Cr:Si in Nb_4_CrSiB_2_, respectively. Figure 3d shows the measured XRD pattern (black cross symbols) of the sample resulting from the initial elemental ratios of 4:1:1:2 for Nb:Cr:Si:B, respectively, together with the calculated patterned produced from the Rietveld refinement analysis (red line), and the difference between them shown as a blue line. The weight percentage of the desired phase, Nb_4_CrSiB_2_, was ≈68 wt % and the remainder was comprised of the phases CrB_2_, Nb_2_Cr_4_Si_5_, Cr_3_B_4_, and NbB. The *a* and *c* lattice parameters obtained from the refinement are 6.109(1) and 11.547(2) Å, respectively, and these values are within 1% of the theoretically predicted values shown in Table S6. The XRD pattern of the full 2θ range along with its refinement can be found in Figure S3c and the detailed results of the refinement are found in Tables 1, S2, and S5.

Both lattice parameters *a* and *c* (Table S5) increase as the size of the major transition metal, M, increases, going from the smaller atoms Mo and W to the larger Nb. In addition, when comparing the lattice parameters, *a* and *c* (Table S5) of the *o*-MAB phases Mo_4_CrSiB_2_, W_4_CrSiB_2_, and Nb_4_CrSiB_2_, with their ternary counterparts, Mo_5_SiB_2_, W_5_SiB_2_, and Nb_5_SiB_2_, a noticeable reduction of the lattice parameters is shown for the *o*-MAB phases due to the replacement of M in Wyckoff position 4c with a smaller element, Cr. Although the ternary Cr_5_SiB_2_ is theoretically predicted to be unstable,^33^ this work clearly shows the possibility to incorporate Cr in ternary T2 phases when the A element is Si and the occupied site is 4*c*, to form out-of-plane chemically ordered phases with the chemical formula M_4_CrSiB_2_ (M = Mo, W, Nb).

To better understand why M in M_5_SiB_2_ can be replaced by Cr at the minority site 4*c* but not at the majority site 16*l*, a computational bonding analysis of the COHP was performed for the 24 shortest bonds in each of the structures M_5_SiB_2_, M_4_CrSiB_2_, Cr_4_MSiB_2_, and Cr_5_SiB_2_, where M = Mo, W, or Nb. The COHP of a bond can be described as an energy-weighted DOS-contribution from that specific bond and thus indicate bonding and antibonding states as a function of energy. By integration of the COHP up to the Fermi level, a measure of the bond strength is obtained. A direct comparison of integrated COHP (iCOHP) values between different structures should be done carefully, and the analysis has here been used only to get a qualitative understanding of the bonding characteristics in the different structures. In addition to the structures listed above, the binary and ternary T2 structures Cr_5_B_3_, Cr_4_WB_3_, and W_4_CrB_3_ (i.e., phases of an equivalent structure but different stoichiometry) have also been studied for comparison. Out of these, Cr_5_B_3_ and W_4_CrB_3_ have been realized experimentally,^51,52^ i.e., also in the latter system Cr will reside on the minority site with Mo at the majority site, although the structure with full Cr population, Cr_5_B_3_, is stable.

The resulting total iCOHP values for the 12 shortest bonds of the different structures are shown in Figure S4, with experimentally verified phases marked by a diamond shape. It can be seen that the iCOHP of the various bonds are affected by substitution of Cr at both the 4*c* and 16*l* sites in different ways for different bonds. The most considerable effect of the substitution is seen in the interactions between the majority sites 16*l*, shown in Figure S4h–l, which increases substantially, for a few bonds by ∼100%, by substitution of Cr for Mo, Nb, or W at the 16*l* site. This implies that the bonding between the 16*l* sites is highly important for stability. Considering the additional phases Cr_5_B_3_, Cr_4_WB_3_ and W_4_CrB_3_, a similar trend is seen, with the main difference that Cr_5_B_3_ and Cr_4_WB_3_, out of which the former is stable, has a considerably larger iCOHP for the 16*l*–16*l* interactions compared to the Cr_4_MSiB_2_ phases.

**Figure 4:**
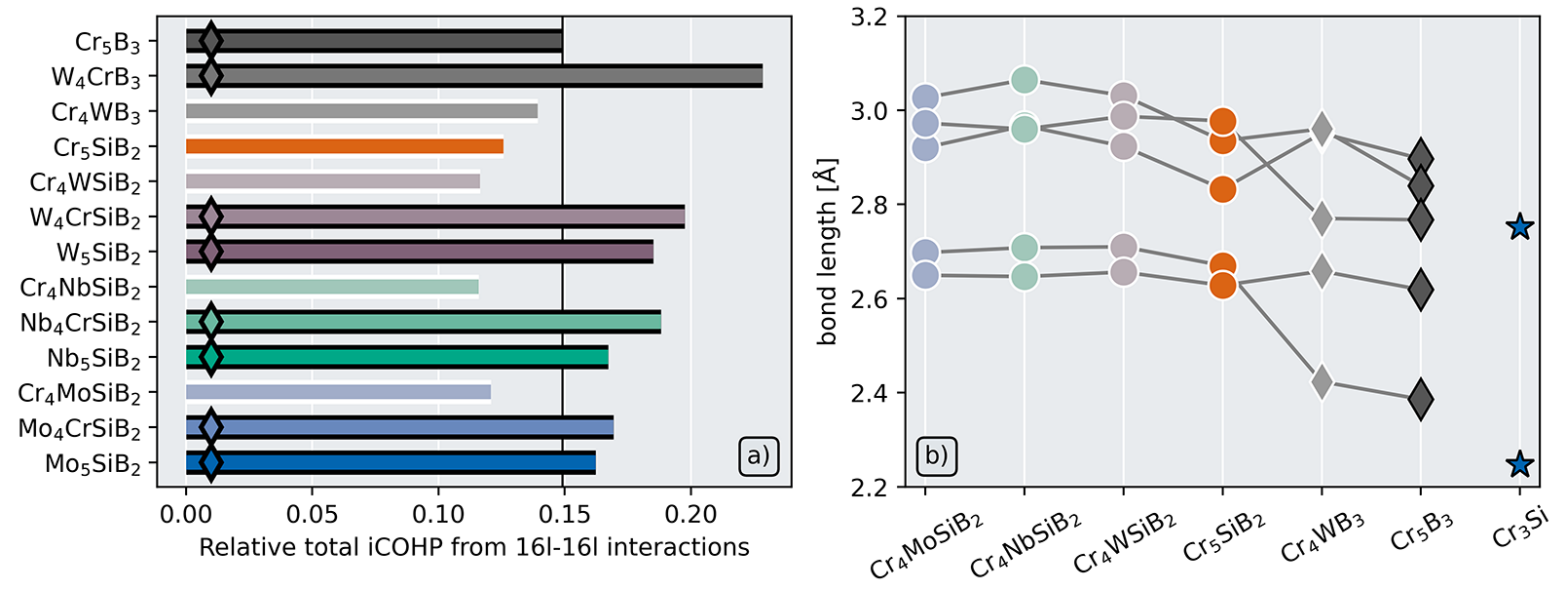
(a) Relative contribution to the total iCOHP for the five shortest 16*l*–16*l* bonds in the T2 structure. Vertical line marks the experimentally stable phase (Cr_5_B_3_) which has the smallest contribution to the total iCOHP from these bonds. (b) Length of the 16*l*–16*l* bonds in T2 structures where the 16*l* sites are populated by Cr. Gray lines link the same bond between the different structures. To the right, the lengths of the two shortest and strongest Cr–Cr bonds in the competing phase Cr_3_Si (experimentally reported) are shown by the blue stars.

In Figure 4a, the relative contribution of the 16*l*–16*l* interactions to the total iCOHP is shown for the different phases. By considering the relative contribution to the total iCOHP rather than the partial iCOHP directly, discrepancies that might be caused by the different structures having different elemental species is avoided. However, a clear trend can still be seen where the 16*l*–16*l* contributions are consistently larger for all the experimentally verified phases than for the seemingly unstable phases.

Figure 4b shows the bond lengths of the 16*l*–16*l* bonds in the six T2 phases with the smallest contributions from the 16*l*–16*l* interactions. These all have Cr populating the 16*l*–16*l* sites, and out of these Cr_5_B_3_, shown in dark gray diamonds, is the only experimentally verified phase, and also the phase with the shortest bond lengths. Along with the T2 phases, one of the competing phases, Cr_3_Si,^32^ is also shown for comparison. It can be seen that this phase has even shorter Cr–Cr bonds. This implies that for the T2 phases with Cr at the 16*l* site, the Cr–Cr bonds becomes too long and therefore too weak to make the structure energetically favorable.

## 4 Conclusions

Herein, we report the synthesis and characterization of three chemically ordered Cr-based T2 MAB phases, establishing out-of-plane ordered MAB phases (*o*-MAB) as a family of materials. Clear evidence for out-of-plane ordering in Mo_4_CrSiB_2_, W_4_CrSiB_2_, and Nb_4_CrSiB_2_ is shown through STEM analysis. The relative elemental ratios of M (M = Mo, W, Nb), Cr, and Si for the three phases, measured by EDX, show a ratio close to the ideal molar ratio of 4:1:1. The crystal structure and weight percentage of phases found in the samples synthesized from the ideal molar ratio 4:1:1:2 of M_4_CrSiB_2_ were obtained from the Rietveld refinement of the samples’ XRD patterns. The three samples show the desired phase to be the main phase of an approximate weight percentage of 98 wt % (Mo_4_CrSiB_2_), 90 wt % (W_4_CrSiB_2_), and 68 wt % (Nb_4_CrSiB_2_).

We also present a computational bonding analysis of the herein reported MAB structures along with the related T2 structures M_5_SiB_2_, Cr_4_MSiB_2_, and Cr_5_SiB_2_. This analysis shows that the 16*l*–16*l* bonds are consistently stronger in the experimentally verified structures than in the hypothetical Cr_4_MSiB_2_ and Cr_5_SiB_2_. Further comparisons with the experimentally verified T2 phase Cr_5_B_3_ show that the Cr–Cr bonds are both stronger and shorter in Cr_5_B_3_ than in Cr_4_MSiB_2_ and Cr_5_SiB_2_, implying the increased length of the Cr–Cr bonds in Cr_4_MSiB_2_ and Cr_5_SiB_2_ is rendering these phases energetically unfavorable.

The results presented herein show the incorporation of a new element, Cr, in the ternary T2 MAB phase where A is Si. The study also sheds further light on the structural requirements for the T2 phase to be stable. This opens the door for expanding the chemistry of the T2 family further, which in turn could enhance the property space toward, e.g., Cr-based magnetism and corrosion resistance, while also adding new potential precursors for synthesizing novel 2D materials.

## Supporting Information Available

Synthesis conditions and parameters and chemical composition, microstructure, and crystal structure details (PDF)
