# Supplementary material for: Experimental and Theoretical Investigations of Out-of-Plane Ordered Nanolaminate Transition Metal Borides: M4CrSiB2 (M = Mo, W, Nb)
Source: Inorg Chem. 2023 Mar 29;62(14):5341–7. doi: 10.1021/acs.inorgchem.2c03729 (PMC10091475; doi:10.1021/acs.inorgchem.2c03729)
Supplement: Supplementary file 1 — ic2c03729_si_001.pdf [file ic2c03729_si_001.pdf]

Supplementary Information

Experimental and theoretical investigations of out-of-plane ordered nanolaminate transition metal borides: M_4CrSiB_2 (M = Mo, W, Nb)

Joseph Halim[1*], Pernilla Helmer[1], Justinas Palisaitis[2], Martin Dahlqvist[1], Jimmy Thörnberg[1], Per O. Å. Persson[2], and Johanna Rosen[1*]

[1] *Materials Design Division, Department of Physics, Chemistry and Biology (IFM), Linköping University, SE-58183, Linköping, Sweden*
[2] *Thin Film Physics Division, Department of Physics, Chemistry and Biology (IFM), Linköping University, SE-58183, Linköping, Sweden*

Email: joseph.halim@liu.se; johanna.rosen@liu.se

S1. Synthesis conditions and parameters

Elemental powders, characteristics details are given in Table S1, of each corresponding desired phase were mixed in air using an agate mortar and pestle in the atomic ratios listed in Table S2. After mixing the powders were cold pressed in air for 5 min into 0.5 cm diameter and 1 cm thick disks using a manual hydraulic press with pressure of 70 KPa. The cold pressed disks were placed in alumina crucible which is then placed in a horizontal tube furnace. The furnace was heated and cooled at rate of 5 °C/min under Ar flow of 5 sccm. The soaking temperature and time are listed in Table S2. After cooling, the samples were crushed into powder using mortar and pestle and sieved through a 450-mesh sieve.

Table S1. Source and characteristics of powders used.

Powder	Purity (wt.%)	Mesh number	Source
Boron	> 95%	amorphous powder	Sigma Aldrich, Germany
Chromium	> 99%	< 325 mesh	Sigma Aldrich, USA
Molybdenum	99.95%	3 to 7 µm	Alfa Aesar, Germany
Niobium	99.8%	< 325 mesh	Alfa Aesar, Germany
Tungsten	99.9%	< 325 mesh	Alfa Aesar, Germany
Silicon	99.999%	< 325 mesh	Alfa Aesar, Germany

Table S2. Summary of the starting composition, synthesis parameters, resulting phases and χ^2 from the Rietveld refinement of the XRD data.

Starting composition (atomic ratio)	Soaking parameters		Resulted phases, wt.% from Rietveld refinement of XRD	χ^2 of the Rietveld refinement of XRD
	Temperature (°C)	Duration (min)		
Mo:Cr:Si:B 4:1:1:2	1700	60	98.35(0.6)% Mo_2CrSiB_2, 0.63(0.06)% Mo_2B_5, 1.02(0.10)% CrB_2	7.15
W:Cr:Si:B 4:1:1:2	1700	60	90.21(0.72)% W_4CrSiB_2, 5.07(0.1)% W_2B, 1.25(0.02)% Cr_2B_3, 3.48(0.41)% Cr_5B_3	11.3
Nb:Cr:Si:B 4:1:1:2	1700	60	67.68(1.32)% Nb_4CrSiB_2, 21.15(1.87)% CrB_2, 10.84(0.12)% $Nb_2Cr_4Si_5$, 1.4(0.1)% Cr_3B_4 8.94(0.21)% NbB	6.87

S2. Chemical composition, microstructure, and crystal structure details

Table S3. Elemental atomic percentages* obtained by EDX in SEM of the out-of-plane ordered quaternary borides (*o*-MAB) represented in this work.

Phase	M’	Cr	Si
Mo_4CrSiB_2	68±1	15±1	17±2
W_4CrSiB_2	70±1	16±1	14±1
Nb_4CrSiB_2	67±2	17±1.0	16±1

*The stated errors are the standard deviation from calculating the average elemental ratios obtained from at least 15 particles.

Table S4. Elemental atomic percentages* for Mo, Cr, Si in Mo_4CrSiB_2 phase obtained using EDX in TEM.

Mo	Cr	Si
70±1.0	14±1	16±2

*The stated errors are the standard deviation from calculating the average elemental ratios obtained from 4 particles.

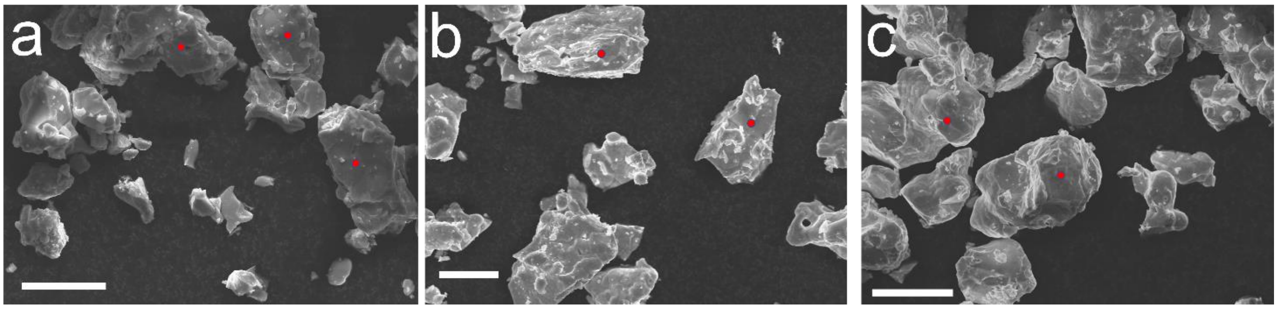

Figure S1. SEM micrographs showing the particles' morphology of the phases (a) Mo_4CrSiB_2, (b) W_4CrSiB_2 and (c) Nb_4CrSiB_2. The red dots represent the particles containing the three elements in M'(Mo, W or Nb)$_4CrSiB_2$, where boron is excluded, according to the EDX point analysis measurements. Boron is not presented in the quantification Table S4 as it is difficult to quantify it using EDX. All scale bars are 10 μm.

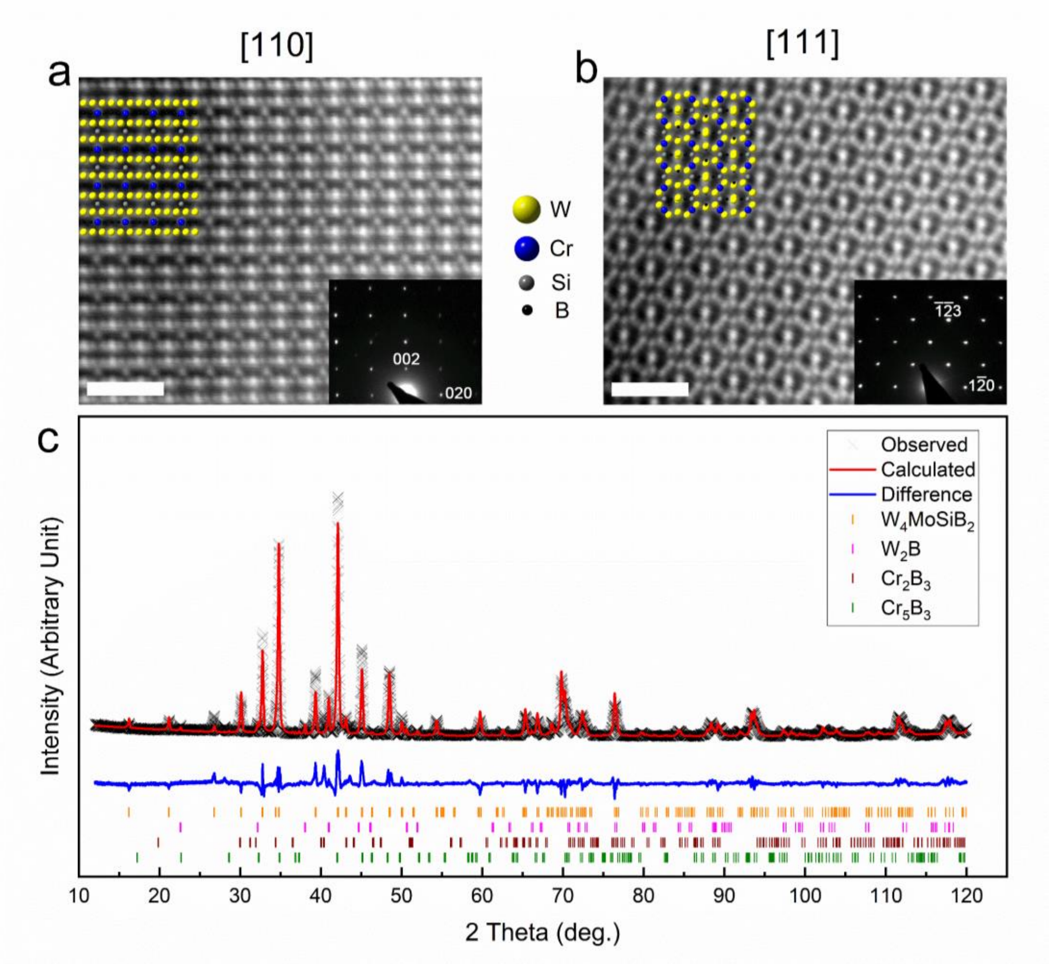

Figure S2. STEM images of W_4CrSiB_2 along the zone axes (a) [110] and (b) [111] with their corresponding SAED as the insets. The crystal structure viewed along both zone axes is overlayed on its respective STEM image. (c) XRD pattern of the W_4CrSiB_2 sample showing the measured pattern (black crosses), Rietveld generated pattern (red line), and the difference between both patterns (blue line). The orange, purple, dark red and green ticks represent the peak positions of the phases W_4CrSiB_2, W_2B, Cr_2B_3 and Cr_5B_3, respectively. Scale bar in (**a**, **b**) is 1 nm.

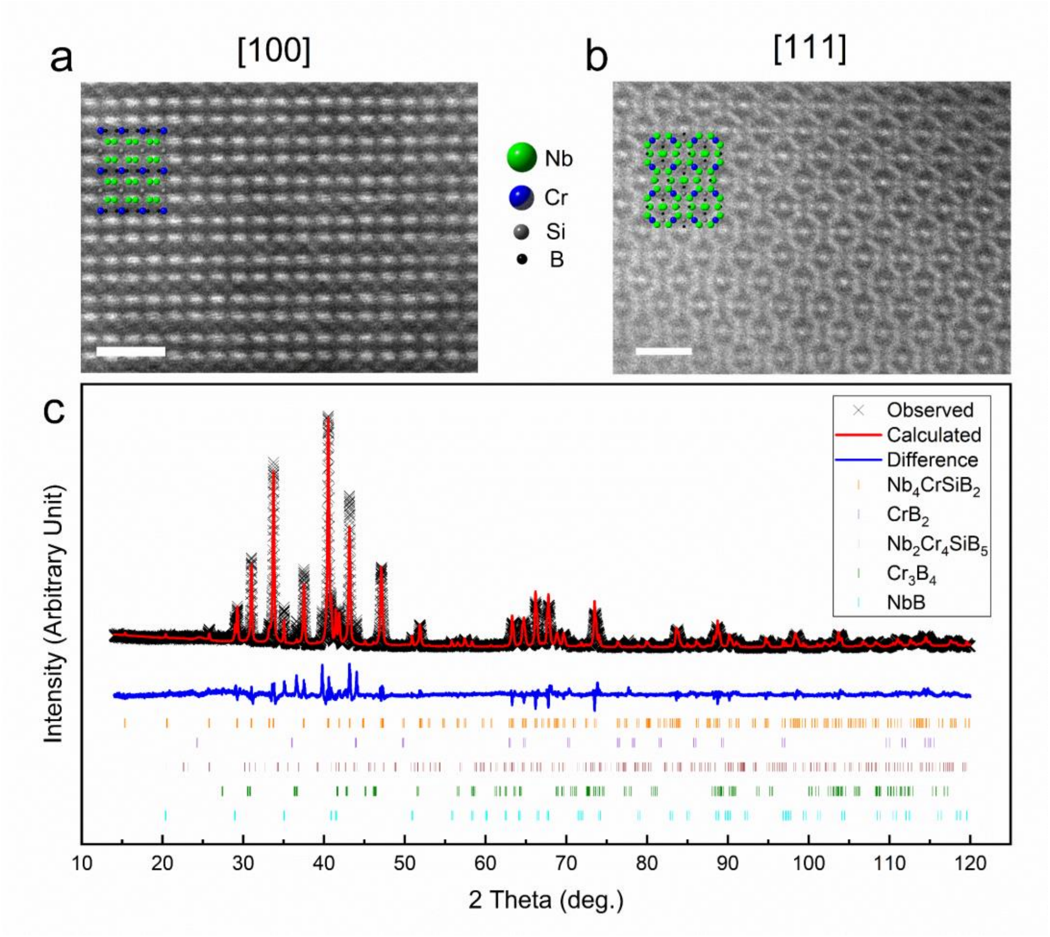

Figure S3. STEM images of Nb_4CrSiB_2 along the zone axes (a) [100] and (b) [111]. The crystal structure viewed along both zone axes is overlayed on its respective STEM image. (c) XRD pattern of the Nb_4CrSiB_2 sample showing the measured pattern (black crosses), Rietveld generated pattern (red line), and the difference between both patterns (blue line). The orange, purple, dark red, green, and turquoise ticks represent the peak positions of the phases Nb_4CrSiB_2, CrB_2, $Nb_2Cr_4SiB_5$, Cr_3B_4 and NbB, respectively. Scale bar in (**a**, **b**) is 1nm.

Table S5. Summary of the LPs and z-coordinates of the out-of-plane ordered quaternary borides (*o*-MAB) obtained herein by Rietveld refinement of the XRD data, and those previously reported of their ternary members.

Phase	*a & b-LP* (Å)	*c-LP* (Å)	Atom coordinates	Ref.
Mo_5SiB_2*	6.0272(3)	11.0671(7)	Mo (*16l*) [0.1641(1) x+1/2 0.13980(1)]<br>B (*8h*) [(0.3784(2) x+1/2 0.0]	[1]
Mo_4CrSiB_2	5.939(5)	11.016(4)	Mo (*16l*) [0.16616(9) x+1/2 0.13855(7)]<br>B (*8h*) [(0.34764(198) x+1/2 0.0]	This work
W_5SiB_2	6.034(3)	11.02(8)		[2]
W_4CrSiB_2	5.942(3)	10.948(3)	W (*16l*) [0.16410(11) x+1/2 0.13927(8)]<br>B (*8h*) [(0.24736(348) x+1/2 0.0]	This work
Nb_5SiB_2*	6.2781((1)	11.654(2)	Nb (*16l*) [0.169 x+1/2 0.1391]<br>B (*8h*) [(0.1156(15) x+1/2 0.0]	[3]
Nb_4CrSiB_2	6.109(1)	11.547(2)	Nb (*16l*) [0.17106(11) x+1/2 0.13813(8)]<br>B (*8h*) [(0.37423(210) x+1/2 0.0]	This work

*Experimentally shown (from neutron diffraction) site mixing of Si and B in both sites: *8h* and *4a*. This has not been investigated in the present study.

Table S6. Computational parameters corresponding to Table S5.

Phase	*a & b-LP* (Å)	*c-LP* (Å)	Atom coordinates
Mo_5SiB_2	6.04227	11.11962	Mo (*16l*) [0.16444 x+1/2 0.13911]<br>B (*8h*) [0.37906 x+1/2 0.0]
Mo_4CrSiB_2	5.92316	11.05407	Mo (*16l*) [0.16776 x+1/2 0.13680]<br>B (*8h*) [0.37656 x+1/2 0.0]
Cr_4MoSiB_2	5.72229	10.45756	Cr (*16l*) [0.16371 x+1/2 0.13964]<br>B (*8h*) [0.38556 x+1/2 0.0]
W_5SiB_2	6.09345	11.04825	W (*16l*) [0.16036 x+1/2 0.14057]<br>B (*8h*) [0.37179 x+1/2 0.0]
W_4CrSiB_2	5.96356	10.97356	W (*16l*) [0.16491 x+1/2 0.13814]<br>B (*8h*) [0.37133 x+1/2 0.0]
Cr_4WSiB_2	5.73129	10.49191	Cr (*16l*) [0.16387 x+1/2 0.13931]<br>B (*8h*) [0.38564 x+1/2 0.0]
Nb_5SiB_2	6.24908	11.65767	Nb (*16l*) [0.16941 x+1/2 0.13743]<br>B (*8h*) [0.38742 x+1/2 0.0]
Nb_4CrSiB_2	6.07975	11.55530	Nb (*16l*) [0.17055 x+1/2 0.13614]<br>B (*8h*) [0.38047 x+1/2 0.0]
Cr_4NbSiB_2	5.78106	10.52061	Cr (*16l*) [0.16187 x+1/2 0.140573]<br>B (*8h*) [0.37179 x+1/2 0.0]
Cr_5SiB_2	5.57008	10.31772	Cr (*16l*) [0.16681 x+1/2 0.13727]<br>B (*8h*) [0.38048 x+1/2 0.0]

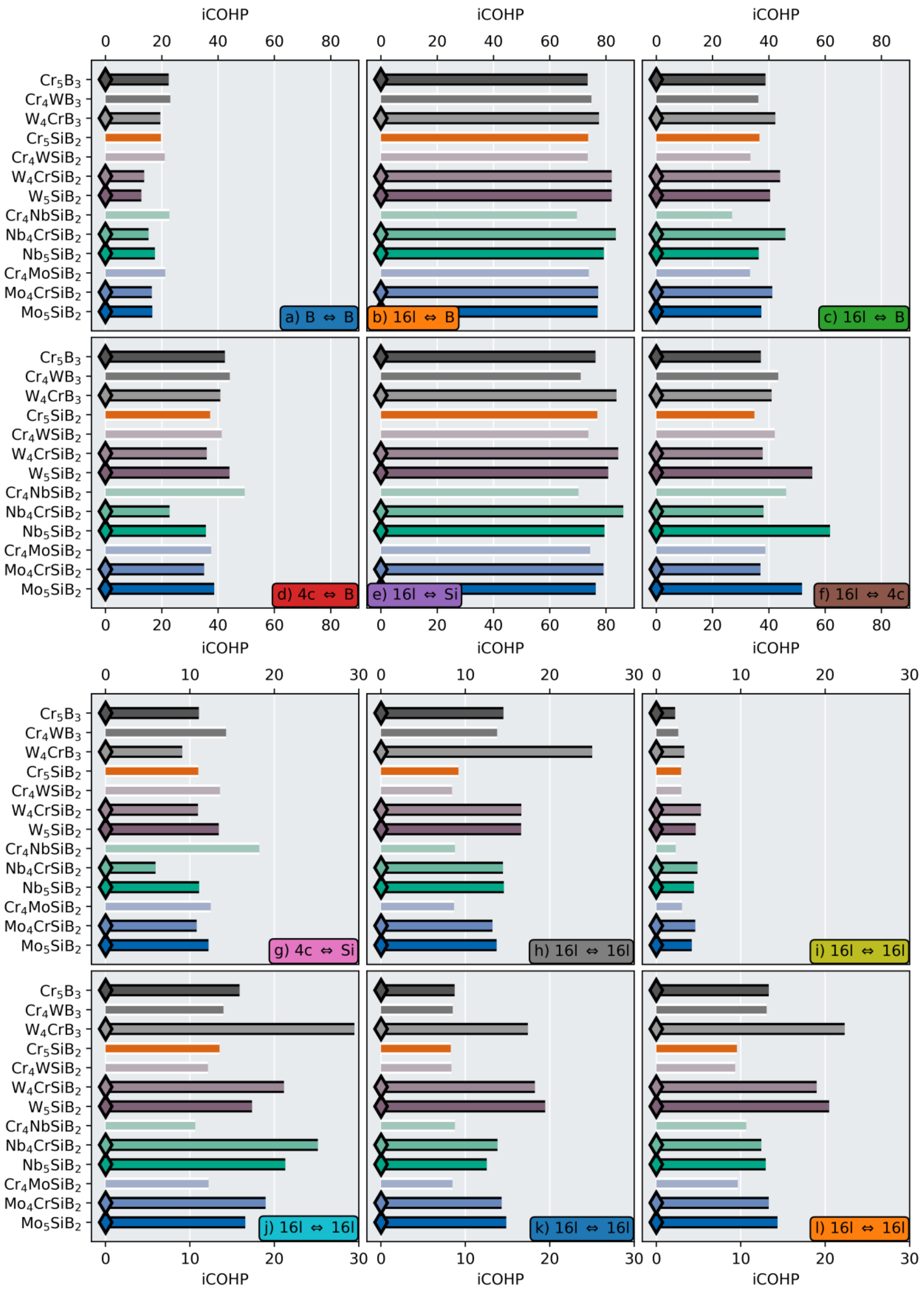

Figure S4. (a)-(l) total iCOHP of the 12 shortest bonds in the considered T2 structures, between sites as specified by each legend. The iCOHPs include the multiplicity of each bond.

References

(1) Rawn, C.; Schneibel, J.; Hoffmann, C.; Hubbard, C. The crystal structure and thermal expansion of Mo_5SiB_2. *Intermetallics* **2001**, *9* (3), 209-216.
(2) Fukuma, M.; Kawashima, K.; Maruyama, M.; Akimitsu, J. Superconductivity in W_5SiB_2 with the T2 phase structure. *Journal of the Physical Society of Japan* **2011**, *80* (2), 024702.
(3) Joubert, J.-M.; Colinet, C.; Rodrigues, G.; Suzuki, P.; Nunes, C.; Coelho, G.; Tedenac, J.-C. The T2 phase in the Nb–Si–B system studied by ab initio calculations and synchrotron X-ray diffraction. *Journal of Solid State Chemistry* **2012**, *190*, 111-117.
